# Targeted NGS on sequential bone marrow biopsies aids in the evaluation of cytopenias and monocytosis and documents clonal evolution—a proof of principle study

**DOI:** 10.1007/s00428-023-03627-1

**Published:** 2023-08-23

**Authors:** Dominik Nann, Achim Rau, Lejla Mahmutovic, Julia Steinhilber, Vanessa Meca, Birgit Federmann, Wichard Vogel, Irina Bonzheim, Leticia Quintanilla-Martinez, Falko Fend

**Affiliations:** 1 Institute of Pathology and Neuropathology, University Hospital Tuebingen and Comprehensive Cancer Center, Tuebingen, Germany; 2 Department of Peptide-Based Immunotherapy and Clinical Collaboration Unit Translational Immunology, Department of Internal Medicine, University Hospital Tuebingen, Tuebingen, Germany; 3 German Cancer Consortium (DKTK) and German Cancer Research Center (DKFZ), Partner Site Tuebingen, Tuebingen, Germany; 4 Cluster of Excellence iFIT (EXC2180) “Image-Guided and Functionally Instructed Tumor Therapies”, Tuebingen, Germany; 5 Department of Hematology, Oncology, Clinical Immunology and Rheumatology, University Hospital Tuebingen, Tuebingen, Germany

**Keywords:** NGS, Bone marrow biopsy, Myeloid neoplasm, Clonal evolution

## Abstract

Differential diagnosis of clonal versus reactive cytopenia and monocytosis, respectively, frequently presents a diagnostic challenge. With the two recent classifications of myeloid disorders, mutational analysis has gained importance as a diagnostic tool. However, reports on its utility on trephine bone marrow biopsies (BMB) are sparse. The aim of our proof of principle study was to determine the suitability of targeted sequencing for the longitudinal evaluation of cytopenia and monocytosis and demonstration of clonal evolution on sequential BMB. Seventy-seven EDTA-decalcified BMB of 33 patients with peripheral cytopenia and/or monocytosis, including at least one follow-up biopsy/patient, were included. Initial morphological diagnoses were idiopathic cytopenia of undetermined significance (ICUS, 8 cases), MDS (without blast increase, 7 cases), MDS with increased blasts/excess blasts (MDS-IB/EB) (11 cases), and CMML (7 cases). Thirty-one genes relevant for myeloid disorders were examined using two custom AmpliSeq NGS panels. Mutations were found in the initial BMB of 5/8 cases of ICUS, thus changing the diagnosis to clonal cytopenia of unknown significance (CCUS), 5/7 MDS, 10/11 MDS-IB/EB, and 7/7 CMML. Clonal evolution was observed in 14/33 (42%) cases, mostly associated with disease progression. None of the wild-type patients acquired mutations during follow-up. NGS-based mutation profiling is a robust diagnostic tool for BMB and provides valuable additional information, especially for cases with no/minimal dysplasia, and for better risk stratification of MDS. Tracking variant allele frequency and appearance of mutations over time allows for observing clonal evolution or relapse.

**Supplementary Information:**

The online version contains supplementary material available at 10.1007/s00428-023-03627-1.

## Introduction

Myelodysplastic syndromes (MDS) are clonal hematopoietic stem cell disorders defined by cytopenia and dysplasia, showing recurrent genetic alterations and an elevated risk of acute myeloid leukemia (AML) [1–4]. It is a disease of elderly people with a median age of 70 years and shows increasing incidence [3, 5–7].

About 90% of cases of MDS exhibit clonal genetic alterations, with mutations recurrently affecting several well-defined classes of genes [8–11]. RNA splicing factors (including *SF3B1*, *SRSF2*, and *ZRSR2*) are mutated in about 60% of cases. *SF3B1* mutations are strongly associated with ring sideroblasts, and *SRSF2* mutations are detected in MDS and about half of cases of chronic myelomonocytic leukemia (CMML) [10–14]. Further recurrent alterations affect epigenetic regulator genes, transcription factors, and genes involved in signaling and the cohesin complex. The most commonly affected genes are *TET2*, *SF3B1*, *ASXL1*, *SRSF2*, *DNMT3A*, and *RUNX1* [9, 10]. The number of mutations increases with disease grade and also during progression, with only 1 to 2 mutations on average in MDS with single lineage dysplasia to 3 to 4 mutations in MDS with excess of blasts (≥5%) [10]. Additionally, MDS patients show recurrent cytogenetic alterations in about half of cases, which also affect prognosis [3, 15–17].

CMML shows myelodysplastic and myeloproliferative features (MDS/MPN) with absolute and relative peripheral blood monocytosis [18] but is otherwise clinically and genetically similar to MDS. The most frequently mutated genes are *TET2* (up to 60%), *SRSF2* (up to 50%), *ASXL1* (40%), *RUNX1* (15%), *NRAS* (11%), and *CBL* (10%) [19–21]. In CMML, recurrent cytogenetic alterations similar to MDS are found in 20–40% of cases [19, 22, 23].

The above-described mutations can also be identified in cases of unexplained cytopenia not fulfilling the diagnostic criteria for MDS, termed clonal cytopenia of undetermined significance (CCUS), if the allele burden is ≥ 2% [11]. Individuals with CCUS carry less mutations per patient on average in comparison to overt MDS [11, 24], but have an elevated risk to develop a myeloid neoplasm in comparison to individuals with idiopathic cytopenia of undetermined significance (ICUS) without mutations [25].

In the absence of cytopenia or monocytosis, the presence of mutations in at least 2% of the nucleated blood cells is called clonal hematopoiesis of indeterminate potential (CHIP). CHIP progresses to an overt hematologic neoplasia at a rate of about 0.5 to 1% per year, depending on clone size, number of mutations, and affected genes [11, 26].

In the two new classifications of myeloid disorders [27, 28], some MDS categories are now defined by mutations. Furthermore, mutational analysis has an important role for the work-up of cytopenia and monocytosis, especially if morphological and cytogenetic findings remain inconclusive. In addition, mutational analysis is an excellent tool for diagnosis of relapse after allogeneic stem cell transplantation (allo-SCT) [29]. Although molecular studies are usually performed on peripheral blood or bone marrow aspirates, BMB sometimes are the only available material. The feasibility of molecular analysis on ethylene diamine tetra-acetic acid (EDTA)-decalcified BMB has been demonstrated in few previous studies [30–34]. In some of these, fresh aspirates were compared with formalin-fixed BMB, with a concordance rate of 96.7 to 100% and with virtually no failure [31, 32, 34]. In the largest study with 192 BMB, only 3.6% of samples failed in sequencing [30]. In addition to mutations, fusion transcripts can be detected with very high reliability in BMB [35].

In particular, for retrospective studies of patients with long follow-up, archival BMB offers a unique opportunity to study the potential diagnostic impact of mutational analysis in patients with unexplained cytopenia and to investigate clonal evolution.

The aim of this proof of principle study was, therefore, to retrospectively perform a targeted mutational analysis of trephine BMB of a cohort of patients, who had been evaluated for cytopenia and/or monocytosis and for whom at least two consecutive biopsies were available for evaluation.

## Material and methods

### Patient samples

Thirty-three patients who had undergone BMB for the evaluation of cytopenia and/or monocytosis and for whom consecutive BMB from different time points were available for review and genetic studies were included in the study. Cases with acute leukemia, lymphoid neoplasms, and obvious reactive causes for cytopenia in the initial biopsy were excluded. The BMB samples were collected from the Institute of Pathology, University Hospital of Tübingen (Germany). Three patients were evaluated for the presence of a therapy-related myeloid neoplasm following chemotherapy for low-grade lymphoma (A2, B3) or myxoid liposarcoma (C9) 6 to 11 years prior. In nine patients (A7, A8, C2, C4, C5, C7, C8, C11, D1), follow-up biopsies after allo-SCT were also investigated. Eight of these 9 (89%) patients experienced disease relapse between one and 123 months after allo-SCT (median 34.5 months). All samples were reviewed by two of the authors (DN and FF) and reclassified according to the 5^th^ Edition of the World Health Organization (WHO) classification and the 2022 International Consensus Classification (ICC) [27, 28]. For the study, MDS was subdivided into “MDS” including MDS of all types with blast count < 5% in the BMB (WHO 5^th^ Edition: MDS with low blasts (MDS-LB), ICC: MDS, not otherwise specified (NOS)) and into “MDS-IB/EB” including MDS of all types with blast count 5–19% (WHO: MDS with increased blasts (MDS-IB1 and MDS-IB2), ICC: MDS with excess blasts (MDS-EB) and MDS/AML). All BMB had been formalin-fixed, decalcified in EDTA for 7–12 hours and embedded in paraffin. Stains for H&E, Giemsa, and NASD chloroacetate esterase as well as iron and reticulin stains and immunohistochemical staining for CD34, CD117, CD61, and CD71 had been performed as part of the initial diagnostic work-up. Immunohistochemistry was done on the Ventana Ultra automated staining system (Ventana Medical Systems, Tucson, AZ, USA) using Ventana reagents, according to the manufacturer’s protocol. The study was conducted in accordance with the Declaration of Helsinki and was approved by the local Ethics Review Committee (106/2013BO2).

### Next-generation sequencing

Targeted mutation analysis was performed by next-generation sequencing on the Ion GeneStudio S5 prime (Thermo Fisher Scientific, Waltham, MA, USA) using an AmpliSeq Custom Panel (AmpliSeq Designer, Thermo Fisher Scientific) encompassing the hotspot regions of 21 genes and the complete coding sequence of 10 genes (see supplemental table 2) frequently mutated in MDS and other chronic myeloid disorders. A second, smaller panel including the hotspot regions of 12 genes and the complete coding sequence of 5 genes was used in a subset of samples (see supplemental table 3). For more detailed information on DNA extraction and integrity, sequencing, validation, statistical analysis, and visualization, see supplemental methods.

## Results

### Clinical and morphological findings

The patients’ basic information is summarized in Table 1. A total of 33 patients with 77 samples were included in the study, of which 27 were males and 6 females with a median age of 63 years (range, 36–84 years). At least two consecutive biopsies were available for all patients and three or more biopsies in 9/33 cases (27%).

**Table 1:** Clinical finding of the patients in total and subdivided to the different groups

	Total	ICUS	MDS	MDS-IB/EB	MDS/MPN
Patients	33	8	7	11	7
Male:female (ratio)	27:6 (4.5)	5:3 (1.67)	7:0	8:3 (2.67)	7:0
Median age (range) [years]	63 (36–84)	55 (36–75)	68 (58–84)	65 (44–77)	66 (55–75)
Samples	77	20	15	25	17
Cases with ≥ 2 follow-up samples	9 (27%)	2 (25%)	1 (14%)	3 (27%)	3 (43%)
Median follow-up (range) [months]	38 (2–130)	61.5 (14–110)	38 (3–73)	44 (5–130)	14 (2–47)
Median leucocytes (range) [/μl] [number of cases with available date]	3890 (750–45,770) [31]	3695 (1100–9000) [8]	4200 (2700–6300) [7]	2830 (750–14,820) [11]	7080 (4410–45,770) [5]
Median hemoglobin (range) [g/dl] [number of cases with available date]	10.5 (6.0–16.3) [30]	11.3 (6.8–14.1) [7]	9.5 (8.1–13.0) [7]	9.2 (6.0–13.2) [11]	13.8 (10.8–16.3) [5]
Median thrombocytes (range) [1000/μl] [number of cases with available date]	133 (5–389) [31]	153.5 (71–389) [8]	88 (37–301) [7]	79 (5–297) [11]	83 (33–202) [5]

*ICUS* idiopathic cytopenia of undetermined significance, *MDS* myelodysplastic syndrome, *MDS-IB/EB* myelodysplastic syndrome with increased blasts/excess blasts, *MPN* myeloproliferative neoplasm

The consensus diagnosis after review of the initial biopsies and before mutational analysis was ICUS in 8 cases (24%, Group A), MDS in 7 cases (21%, Group B), MDS-IB/EB in 11 cases (33%, Group C), and CMML or MDS/MPN-U (1 patient) in 7 cases (21%, Group D). Of note, all patients with an initial diagnosis of ICUS were subsequently diagnosed with a myeloid neoplasm during follow-up (see below).

### Mutational landscape

All cases were analyzed by NGS and had a DNA integrity of ≥ 200 base pairs. The mean average read depth of the NGS sequence analysis was 3951 reads for panel 1 (range, 669–8934) and 4007 reads for panel 2 (range, 633–14,583). For panel 1, in median 4 amplicons (0.8%) were with less than 50 reads (range, 4–14 (0.8–3.1%)) and in median 5.5 amplicons (1.2%) with less than 200 reads (range, 4–34 (0.8–7.4%)). For panel 2, in median 3 amplicons (1.2%) were with less than 50 reads (range, 0–9 (0–3.6%)) and in median 9 amplicons (3.6%) with less than 200 reads (range, 3–27 (1.2–10.9%)).

The median time between biopsy procurement and NGS analysis was 2.1 years, with a maximum of 18.4 years.

**Fig. 1:**
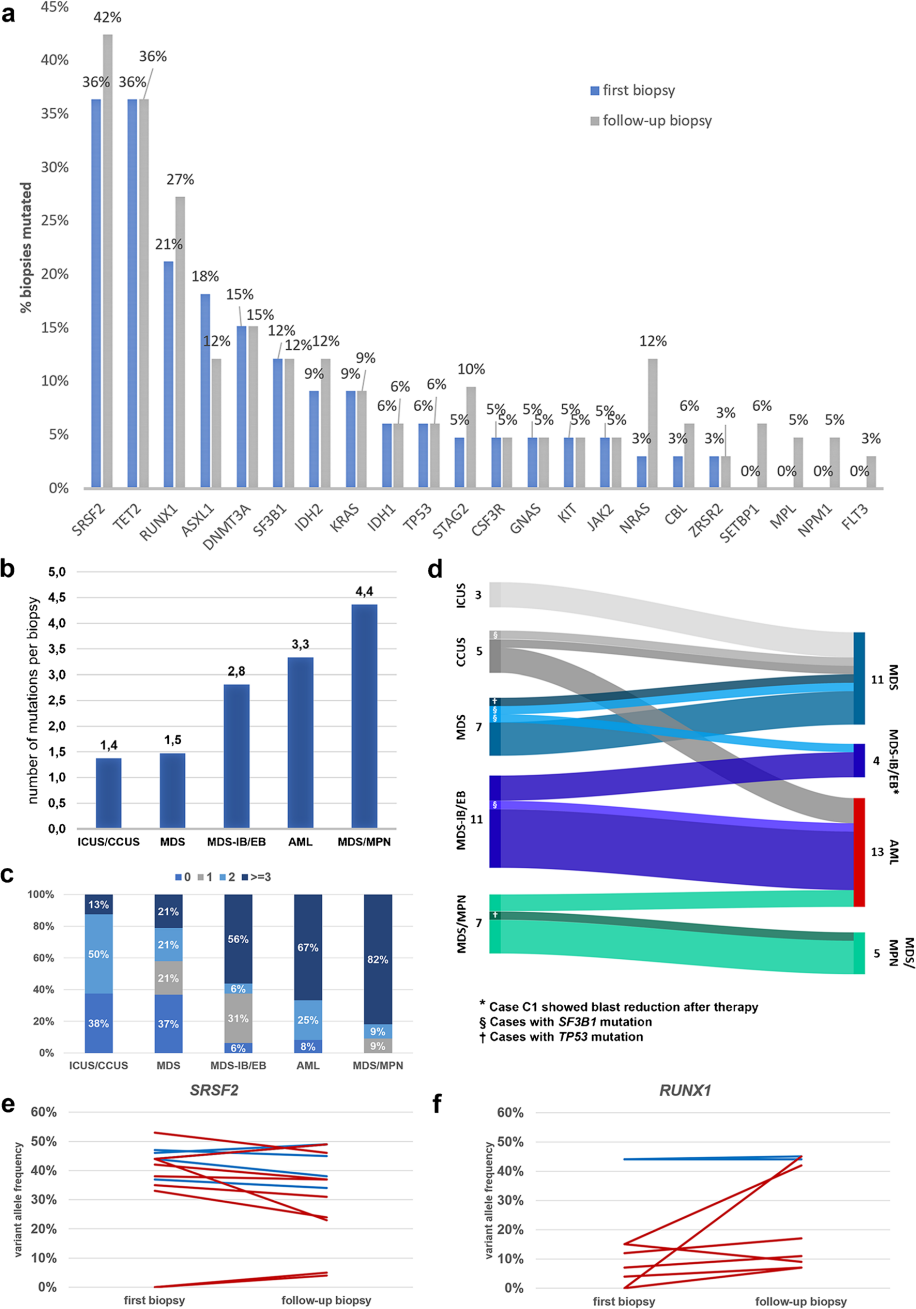
Mutation frequencies and clonal evolution. **a** Mutational spectrum and mutation frequencies shown separately for the first and the follow-up biopsy with maximum progression of disease. **b** Mean number of mutations per diagnostic group. **c** Distribution of the number of mutations per case for the different diagnostic groups. **d** Changes in diagnostic classification over time due to disease evolution from the initial biopsy to the follow-up biopsy with maximum progression of disease. In brighter colors are the cases with *SF3B1* mutation and in darker the cases with *TP53* mutation. **e**, **f** Variant allele frequency course of two exemplary genes from the first biopsy to the follow-up biopsy with maximum progression of disease. Blue: MDS cases, red: MDS-IB/EB cases

In total, 207 mutations were found. The most frequently mutated genes were *SRSF2* and *TET2* with 36% in the initial biopsy (Fig. 1A), followed by *RUNX1* (21%), *ASXL1* (18%), *DNMT3A* (15%), *SF3B1* (12%), *IDH2*, *KRAS* (both with 9%), *IDH1*, *TP53* (all with 6%), *STAG2*, *CSF3R*, *GNAS*, *KIT*, *JAK2* (all with 5%), *NRAS*, *CBL*, and *ZRSR2* (all with 3%). The mutation frequencies of ten genes increased in follow-up biopsies (*SRSF2*, *RUNX1*, *IDH2*, *STAG2*, *NRAS*, *CBL*, *SETBP1*, *MPL*, *NPM1*, and *FLT3*). The frequencies for *STAG2* and *NRAS* doubled and increased fourfold, respectively, but this failed to statistical significance. In three cases, cytogenetic progression was documented over time (see supplemental table 1).

The median variant allele frequencies (VAF) for the most frequently mutated genes were 44% (range, 33–53%) for *SRSF2*, 44.5% (range, 3–75%) for *TET2*, and 15% (range, 4–44%) for *RUNX1* (supplemental table 1).

Overall, mutations were identified in 22/31 examined genes. The average number of mutations depended on disease category and increased over time (Fig. 1B, C). While most CCUS and MDS showed one or two mutations (mean 1.4 and 1.5 mutations, respectively), over 50% of MDS-IB/EB exhibited two or more mutations (mean 2.8) and CMML mostly showed three or more mutations (mean 4.4).

The comparison of mutated genes between MDS and MDS-IB/EB cases showed that only *IDH2* was significantly higher in the MDS-IB/EB group (*p* = 0.0212). Changes in VAFs between the initial and follow-up biopsy for individual patients are shown for two commonly mutated genes (*SRSF2* and *RUNX1*) in Fig. 1E, F. *SRSF2* showed no clear trend in VAFs in MDS (median 44% (first) vs. 45% (follow-up)) or MDS-IB/EB cases (median 38% vs. 31%). The VAFs of *RUNX1* remained unchanged in the two MDS cases (median 44% (first) vs. 44.5% (follow-up)) and showed an increase in MDS-IB/EB cases (median 7% vs. 11%).

### Group A—ICUS at presentation

**Fig. 2:**
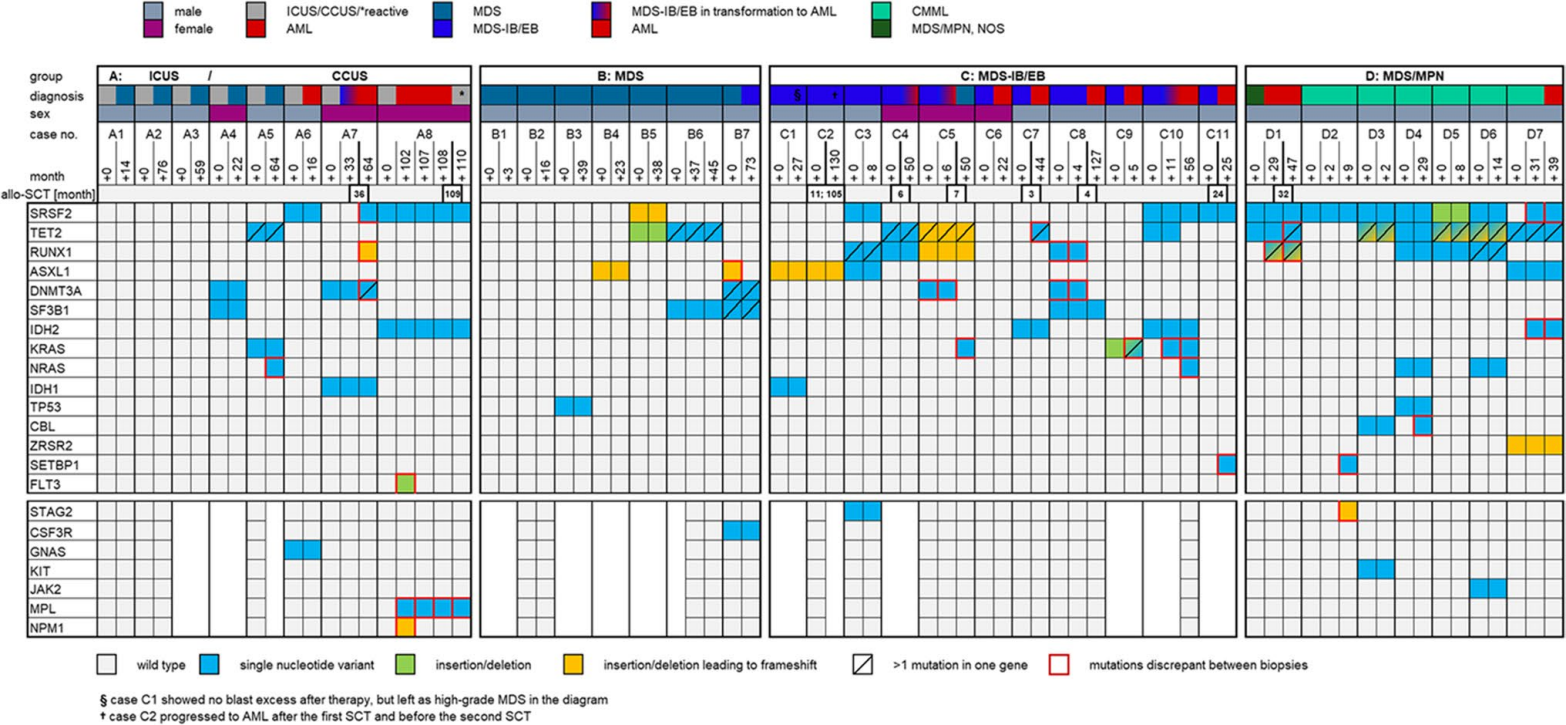
Mutational profiles according to diagnostic group. The chart shows the mutational profile for each group. For each case, the consecutive biopsies are next to each other from left to right, illustrating the evolution of diagnosis and mutational spectrum. Only genes mutated in at least one sample are shown; the genes with only wild-type results (*BRAF*, *CALR*, *CEBPA*, *ETNK1*, *HRAS*, *IKZF1*, *STAT3*, and *U2AF1*) are not depicted. Four of five biopsies from patient A8 and one biopsy from patient C10 were additionally analyzed by the commercially available Oncomine Myeloid Research Panel (Thermo Fisher Scientific, Waltham, MA, USA) during routine processing

**Fig. 3:**
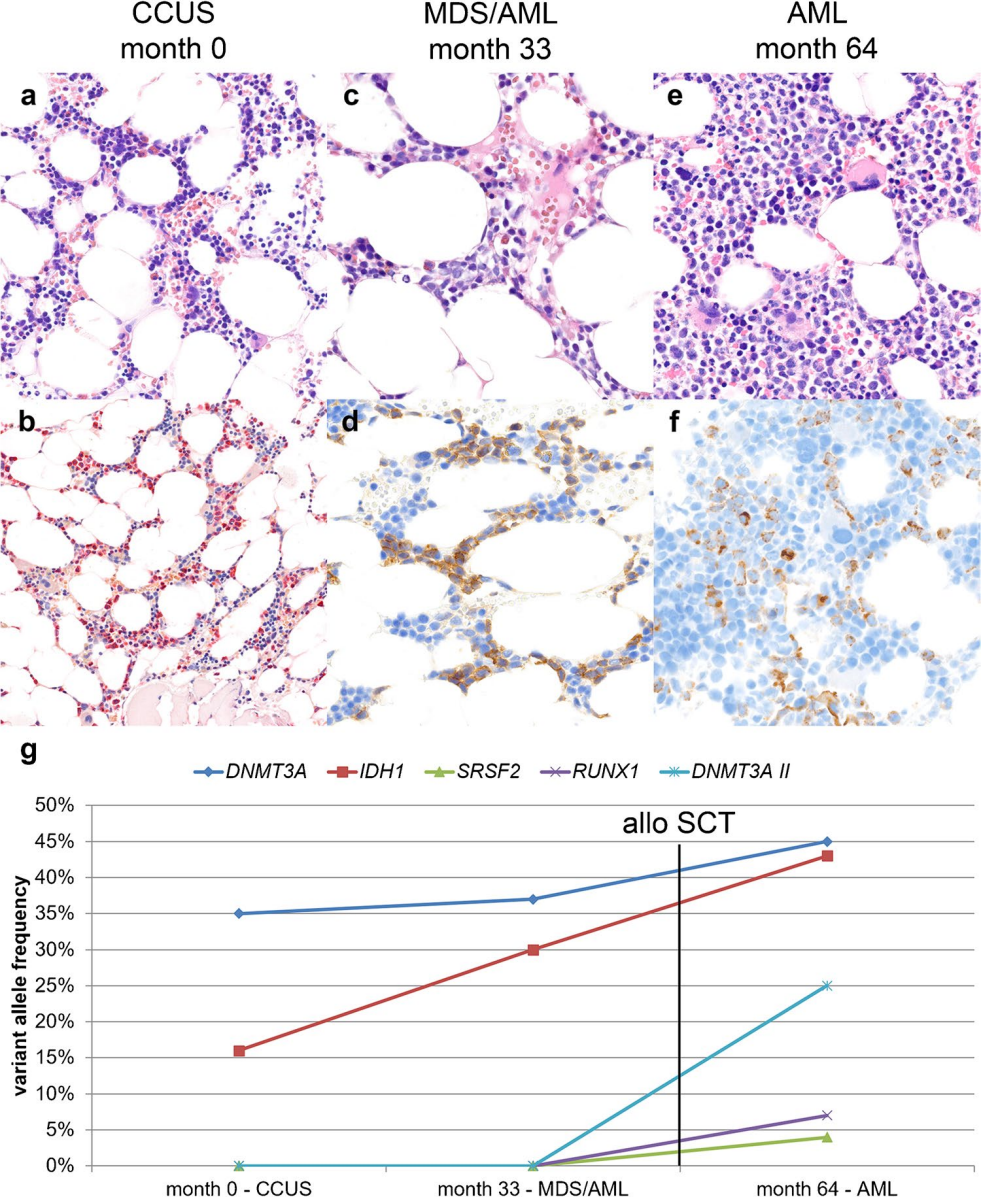
Morphological and genetic evolution of case A7. A 55-year-old woman evaluated for cytopenia. She developed MDS with excess of blasts in transformation to AML 33 months later and underwent allo-SCT, but relapsed 26 months after allo-SCT with AML. **a**, **b** Biopsy month 0; **a** normocellular bone marrow (BM) biopsy without evidence of dysplasia and normal cytogenetics (hematoxylin and eosin (HE), 400× original magnification); **b** usual distribution of granulopoiesis (in red) and erythropoiesis demonstrated by naphthol-AS-D-chloroacetate esterase reaction (200×). **c**, **d** Biopsy month 33; **c** increase in middle-sized, interspersed blasts, diagnosed as MDS with excess of blasts in transition to AML (HE, 400×); **d** the blasts are positive for CD117 (immunoperoxidase, 400×). **e**, **f** Biopsy month 64 and after allo-SCT; **e** hypercellular BM with many blasts (HE, 400×), which are positive for CD34 (**f** immunoperoxidase, 600×). **g** Variant allele frequencies (VAF) of detected mutations in consecutive biopsies show in the first biopsy two mutations in *DNMT3A* and *IDH1* with a VAF of 35 and 16%, respectively. The second biopsy reveals the same mutational spectrum, but with increased VAFs and after allo-SCT there are three more mutations in *SRSF2* (4%), *RUNX1* (7%), and *DNMT3A* (25%), and the known mutations also increased (*DNMT3A* 45%, *IDH1* 43%)

**Fig. 4:**
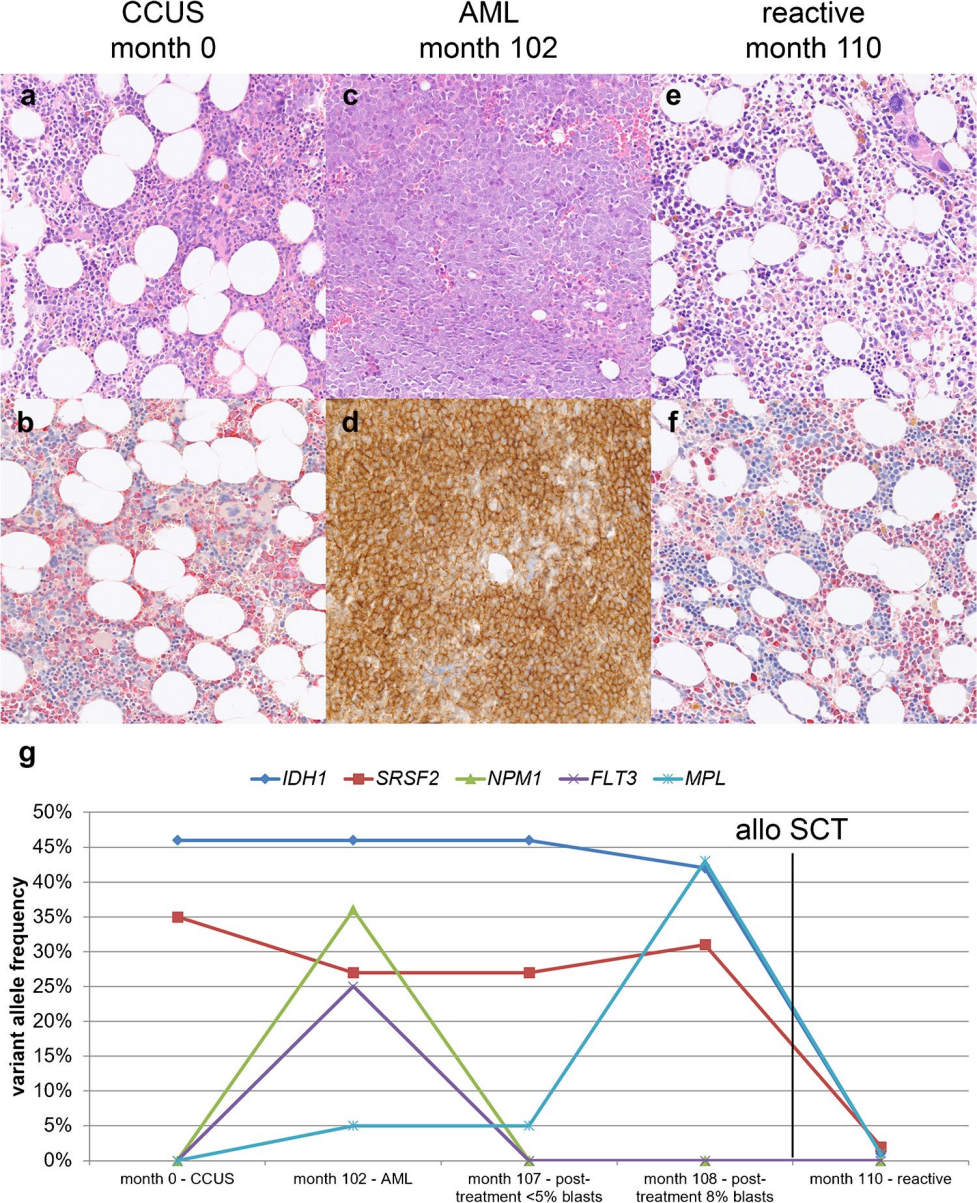
Morphology and genetic evolution of case A8. A 54-year-old woman with inconspicuous cytogenetics and reactive bone marrow (BM) at the first biopsy. One hundred and two months later, she was diagnosed with AML and after therapy the BM showed a blast count of <5% and 8%, respectively. After allo-SCT, there were no morphological residues of the AML, compatible with reactive changes. **a**, **b** Biopsy month 0; **a** normocellular BM biopsy with reactive changes, but without apparently dysplastic changes (hematoxylin and eosin (HE), 200× original magnification); **b** usual distribution of granulopoiesis and erythropoiesis demonstrated by naphthol-AS-D-chloroacetate esterase reaction (200×). **c**, **d** Biopsy month 102; **c** diffuse infiltrates by an acute myeloid leukemia with lots of blasts (HE, 200×) and positivity for CD33 (**d** CD33 immunoperoxidase, 200×). **e**, **f** Biopsy month 110; **e** normocellular BM biopsy with reactive changes with increased and left shifted erythropoiesis and left shifted granulopoiesis (HE, 200×), also demonstrated by naphthol-AS-D-chloroacetate esterase reaction (**f** 200×). **g** Variant allele frequencies (VAF) of mutations in consecutive biopsies. The first biopsy shows an *IDH2* (VAF 46%) and a *SRSF2* mutation (35%). The AML reveals additional mutations of *NPM1* (36%), *FLT3* (25%), and *MPL* (5%). After therapy, *IDH2* and *SRSF2* mutations do not decrease, but *NPM1* and *FLT3* cannot be detected anymore, only *MPL* remains. One month later, the *MPL* mutation increases to 43%. After allo-SCT, the three mutations in *IDH2*, *SRSF2*, and *MPL* are still detectable with low frequencies (1, 2, and 1%, respectively), demonstrating a small amount of residual mutated cells after transplantation

This group included 8 cases (Fig. 1D and Fig. 2), five men and three women with a median age of 55 years (range, 36 to 75 years). The follow-up was between 14 and 110 months (median 61.5 months). Six cases had two evaluable biopsies, one case each three and five biopsies, respectively. Three cases (A1 to A3) were wild type for all investigated genes, whereas five cases (A4 to A8) showed at least one mutation in the first biopsy. None of the three wild-type cases acquired a mutation during follow-up, although a consensus diagnosis of MDS was rendered in the follow-up biopsy. All three wild-type cases also had normal cytogenetics. Of note, all confirmed cases of CCUS progressed to MDS (A4, A5) or AML (A6 to A8). The two cases progressing to MDS showed *DNMT3A* and *SF3B1* (A4) and *TET2* and *KRAS* (A5) mutations in the first biopsy with acquisition of an additional *NRAS* mutation in case A5. Two (A7 and A8) of three cases with AML progression are detailed in Figs. 3 and 4.

### Group B—MDS at presentation

Group B included seven males with an age of 58 to 84 years (median 68 years) and a follow-up between three and 73 months (median 38 months). Morphologically, all patients were classified as MDS in the first biopsy and only one patient progressed to an MDS-IB/EB in the second biopsy (B7). Two cases (B1 and B2) lacked detectable mutations in both biopsies and also showed normal cytogenetics. The four cases with mutations and stable disease had one to three mutations (median 1.5 mutations per case) and no additional mutations in the subsequent biopsies. The single case with progression (B7) had mutations in *SF3B1*, *DNMT3A*, *ASXL1*, and *CSF3R*.

### Group C—MDS-IB/EB at presentation

Group C contained 11 patients (M:F = 8:3) with MDS-IB/EB, aged 44 to 77 years (median 65 years). The follow-up time was between five and 130 months (median 44 months). In three patients, three biopsies were analyzed. In six patients (C2, C4, C5, C7, C8, C11), also post allo-SCT biopsies were investigated. Nine patients (82%) progressed to AML (C2, C4 to C11), five with AML progression after SCT (C2, C4, C7, C8, C11), three cases with progression without previous SCT (C6, C9, C10), and one case with an AML before SCT and relapse as MDS after SCT (C5). Case C1 showed a decreased blast count after azacitidine therapy (27 months) and case C3 remained stable. Only one case (C6) with an initial karyotype of 46, XX; del(5q), del(17) remained wild type for all investigated genes despite progression to AML. The two cases without AML progression (C1 and C3) showed a stable genotype over time. The number of mutations remained stable in five cases (5/10, 50%, C1 to C5) and increased in four cases (4/10, 40%, C7, C9 to C11). Despite the frequent progression to AML, the overall number of mutations only slightly increased in this group. Some cases showed evidence of divergent clonal evolution during progression or in relapse after allo-SCT, but all showed evidence of clonal relationship. In case C8, the AML relapse after allo-SCT shared only the *SF3B1* mutation, whereas the *RUNX1* and the *DNMT3A* mutation disappeared. Case C5 showed disappearance of the *DNMT3A* mutation and a new *KRAS* mutation in the third biopsy after allo-SCT. In case C10, two mutations (*SRSF2* and *IDH2*) remained during progression, whereas a *TET2* mutation disappeared and a *KRAS* mutation and a *NRAS* mutation were newly acquired.

### Group D—MDS/MPN at presentation

This group contained 6 males with CMML and 1 with MDS/MPN, NOS with a median age of 66 years (range, 55 to 75 years). The follow-up time was between 2 and 47 months (median 14 months). Four of seven patients (57%) remained stable, two cases progressed to AML, and one patient from CMML-1 to CMML-2 (D6). The mutational profile was distinct from the other groups, with *SRSF2* mutations in all cases (100%), *TET2* in six cases (86%), and *RUNX1* in four cases (57%). *JAK2*, *KIT*, *ZRSR2*, and *CBL* mutations were only identified in this group. In four cases (D3, D5 to D7), all samples had at least two *TET2* mutations, and in one case (D1), the last sample revealed a second mutation.

### MDS following chemotherapy

Three patients described above had a history of chemotherapy as outlined above. Given the low number of cases, no evident differences to the other cases were observed.

## Discussion

The present study demonstrates the feasibility of targeted mutational analysis in patients with MDS, MDS/MPN, and ICUS, using EDTA-decalcified bone marrow biopsies as alternative DNA source. By focusing on cases with sequential biopsies and known outcome, we could show that mutational analysis can aid in the evaluation of cases with cytopenia and monocytosis of unknown cause and improve risk stratification. Furthermore, the reliable identification of identical mutations in follow-up biopsies—in addition to the demonstration of clonal evolution—served as internal validation and highlights the robustness of the technique in BMBs. Given the increasing importance of mutational profiling for risk assessment in MDS, which has recently led to the proposal of a molecular international prognostic scoring system for MDS (IPSS-M) [36], the use of archival BMB for mutational profiling is an opportunity for long-term retrospective studies.

Despite the small sample size, the mutational landscape showed similarities, but also differences in the overall distribution of the detected mutations in comparison to large published studies [9, 10]. The six most mutated genes (*SRSF2*, *TET2*, *RUNX1*, *ASXL1*, *DNMT3A*, and *SF3B1*) are identical to larger studies, but with different frequencies. The high frequencies of *SRSF2* hotspot and *RUNX1* mutations are due to the inclusion of CMMLs in our study, with *SRSF2* being one of the most commonly mutated genes in CMML and *RUNX1* also showing a higher frequency in CMML as compared to MDS [14, 19–21].

One of the aims of our retrospective study was to evaluate whether mutational analysis in cases of cytopenia without sufficient evidence for MDS but later development of a myeloid neoplasm might have helped to predict this evolution. Of the 8 cases initially diagnosed as ICUS (Group A), 3/5 cases reclassified as CCUS due to the presence of mutations in the first biopsy progressed to MDS-IB/EB or AML, whereas the remaining 2 mutated cases and the 3 cases without detectable mutations from this group advanced to MDS without blast increase. The cumulative risk of evolution to myeloid neoplasm increases from ICUS, over CCUS with low to highly predictive mutation pattern [25]. The highly predictive pattern contains mutations in spliceosome genes (*SF3B1*, *SRSF2*, *U2AF1*) and mutations in *TET2*, *DNMT3A*, or *ASXL1*, in addition to other mutations. In accordance, all our CCUS patients had a highly predictive mutation pattern in the first biopsy. This observation suggests that mutational profiling in cytopenia without fulfilling morphological criteria for MDS may help to select patients which require closer follow-up. In line with the literature, the cases remaining ICUS were younger than the CCUS cases (median 48 years vs. 55 years) [11, 24, 25]. The 3 ICUS cases, which despite the persistent lack of mutations nevertheless progressed to MDS, together with the two cases of non-mutated, cytogenetically silent MDS highlight the absence of genetic changes in a subset of morphologically defined MDS, approximately 6–10% in recent large cohorts [8–11, 36]. Whether these cases truly represent clonal disorders or mimickers of MDS needs to be shown, since ICUS has a high negative predictive value for the development of MDS [37].

The MDS cases (Group B) showed no progression except for one case, confirming the presence of a subset of stable MDS with little progression risk. Two of five cases showed mutations in *SF3B1*, generally associated with favorable prognosis; however, one case (B7), in addition to *DNMT3A* and *CSF3R* mutations, also exhibited a prognostically unfavorable *ASXL1* mutation and progressed to MDS-IB/EB [3, 38, 39]. The importance of concomitant alterations in MDS with *SF3B1* mutations has been stressed in the recent IPSS-M for MDS [36]. Although the three remaining cases had mutations in genes (*TP53*, *ASXL1*, *SRSF2*) associated with poor risk, they did not show progression during the observation time [3, 38, 39].

Cases in the MDS-IB/EB group (Group C) had more mutations per case compared to groups A and B as expected [9, 10]. Of note, all cases with mutations showed also at least one mutation associated with poor prognosis, especially *RUNX1* in 36% of cases. Only one case lacked mutations, but exhibited a complex karyotype. The results of groups A to C underline the added prognostic information for risk stratification gained by mutational analysis. Whether a molecular risk stratification of MDS [36] will only expand current classification schemes or perhaps replace them in the future, as recently suggested, remains to be seen [40].

In our study, all CMML cases showed a *SRSF2* hotspot mutation, accompanied by *TET2* mutations in all but one case, underlining the strong association of this combination with CMML [14, 19–21]. Progression or transformation to AML (29%) occurred at a similar frequency compared to the literature (15 to 30%) [18]. Except for one patient, all CMML cases showed mutations associated with adverse prognosis [41].

Given the extensive literature on molecular profiling of myeloid neoplasms, published data on mutational analyses performed on decalcified, formalin-fixed, paraffin-embedded (FFPE) BMB or FFPE clot sections are very limited [30–35, 42]. Our study is in line with published data, which showed a very high concordance of the results between paired fresh material and EDTA-decalcified, FFPE BMB and a very high reproducibility between different methods of DNA isolation [31, 32, 34]. This underlines the potential of mutational analysis on trephine BMB, especially for cases without other available sources of DNA and for retrospective analyses. Of note, no cases in this study had to be excluded due to low DNA quality, similar to a failure rate of only 3.6% in previous studies [31, 32, 34].

A single study included a larger number of follow-up biopsies, allowing comparison to our results [33]. Using a 12-gene panel in a cohort of 145 patients including 35 follow-up samples, Bräuninger et al. demonstrated in 6 MDS/AML cases a disappearance (5 cases) or reduction (1 cases) of mutations after successful myeloablative treatment and persistence of the mutations in the 4 remaining cases with evident residual disease. In 16 cases with initially equivocal morphology and available follow-up biopsies, mutations were detected in 13 patients. All 13 developed manifest MDS with persistence of the same mutations in the subsequent biopsies, with additional mutations in two cases. Three patients showed persistent wild-type results for all examined genes in all biopsies and did not progress to MDS [33].

In conclusion, NGS-based mutation profiling is a robust diagnostic tool for EDTA-decalcified BMB. Mutation analysis offers valuable additional information, especially for cases of cytopenia or monocytosis without dysplasia and for better risk stratification of MDS. Tracking variant allele frequency and appearance of mutations over time is a very good tool to observe clonal evolution and detect recurrence and clonal relationship after allo-SCT. Nevertheless, the lack of mutations in some of our cases with confirmed MDS and the common occurrence of CHIP in elderly patients underline the necessity for integration of clinical, morphological, and molecular findings for diagnosis.

### Supplementary information

ESM 1 (DOCX 128 kb)

## Data Availability

The datasets used and/or analyzed during the current study are available from the corresponding author on reasonable request.
