# Supplementary material for: Targeted NGS on sequential bone marrow biopsies aids in the evaluation of cytopenias and monocytosis and documents clonal evolution—a proof of principle study
Source: Virchows Arch. 2023 Aug 23;483(6):835–45. doi: 10.1007/s00428-023-03627-1 (PMC10700460; doi:10.1007/s00428-023-03627-1)
Supplement: Supplementary file 1 — (DOCX 128 kb) [file 428_2023_3627_MOESM1_ESM.docx]

Supplemental Material

**Targeted NGS on sequential bone marrow biopsies aids in the evaluation of cytopenias and monocytosis and documents clonal evolution**

Dominik Nann, Achim Rau, Lejla Mahmutovic, Julia Steinhilber, Vanessa Meca, Birgit Federmann, Wichard Vogel, Irina Bonzheim, Leticia Quintanilla-Martinez, Falko Fend

**Contents**

Supplemental methods 3

Supplemental figure 5

Supplemental tables 6

Supplemental references 18

**Supplemental methods**

**DNA extraction and integrity**

DNA was extracted from 5 μm whole paraffin sections after dewaxing and proteinase K digestion, applying the Maxwell RSC FFPE Plus DNA Kit (AS1720) and the Maxwell RSC instrument (Promega, Mannheim, Germany) according to the manufacturer’s instructions.

The integrity of the DNA was analyzed following the protocol by van Dongen *et al.* [1].

**Next generation sequencing**

Amplicon library preparation and semiconductor sequencing was done according to the manufacturers’ manuals using the Ion AmpliSeq Library Kit v2.0, the Ion Library TaqMan Quantitation Kit on the LightCycler 480 (Roche, Basel, Switzerland), the Ion 510 & Ion 520 & Ion 530 Kit – Chef on the Ion Chef and the Ion 520 Chip Kit (Thermo Fisher Scientific). Output files were generated with Torrent Suite 5.12.0. Variant calling of non-synonymous somatic variants compared to the human reference sequence hg19 was performed using Ion Reporter Software (Thermo Fisher Scientific, Version 5.10). Variants called by the Ion Reporter Software were visualized using the Integrative Genomics Viewer (IGV; Broad Institute, Cambridge, MA, USA; Version 2.8.0) to exclude panel-specific artefacts. The NCBI dbSNP database (including GnomAD, ExAC and TOPMED) was used to exclude SNPs.

**Validation and primer design for targeted re-sequencing using the Ion Amplicon Library Preparation Fusion Method**

For validation of the NGS results, variants with low allelic frequencies or samples with a high level of fixation artefacts were re-analyzed as single amplicons using a targeted re-sequencing approach on the Ion GeneStudio S5 prime. Primers were designed using the primer3 software. The primers were composed of either the A adapter or the trP1 adapter, the barcode sequence and barcode adapter sequence and the target primer sequence. Each gene region was amplified using two primer pairs (A Forward and trP1 Reverse or A Reverse and trP1 Forward) to enable bidirectional sequencing. Library preparation was done according to the manufacturer’s protocol (Thermo Fisher Scientific). For description of primers design see supplemental table 4.

**Statistical analysis and visualization**

Statistical analysis on mutation parameters were conducted using the chi square test. p-values < 0.05 were considered as significant.

For visualization of the data in a Sankey diagram the tool from the homepage https://sankeymatic.com was used.

**Control group**

As control group, we studied 4 BMB of patients with confirmed causes of reactive cytopenia (e.g. vitamin deficiencies). All control cases showed wild type for all examined genes.

**Supplemental figure**

**Figure S1** The diagram shows the different length of time from the initial biopsy to the following biopsy or in some cases biopsies, marked with small lines. The small, red lines indicated the allogenic stem cell transplantations. The different colors mark the different groups.

**
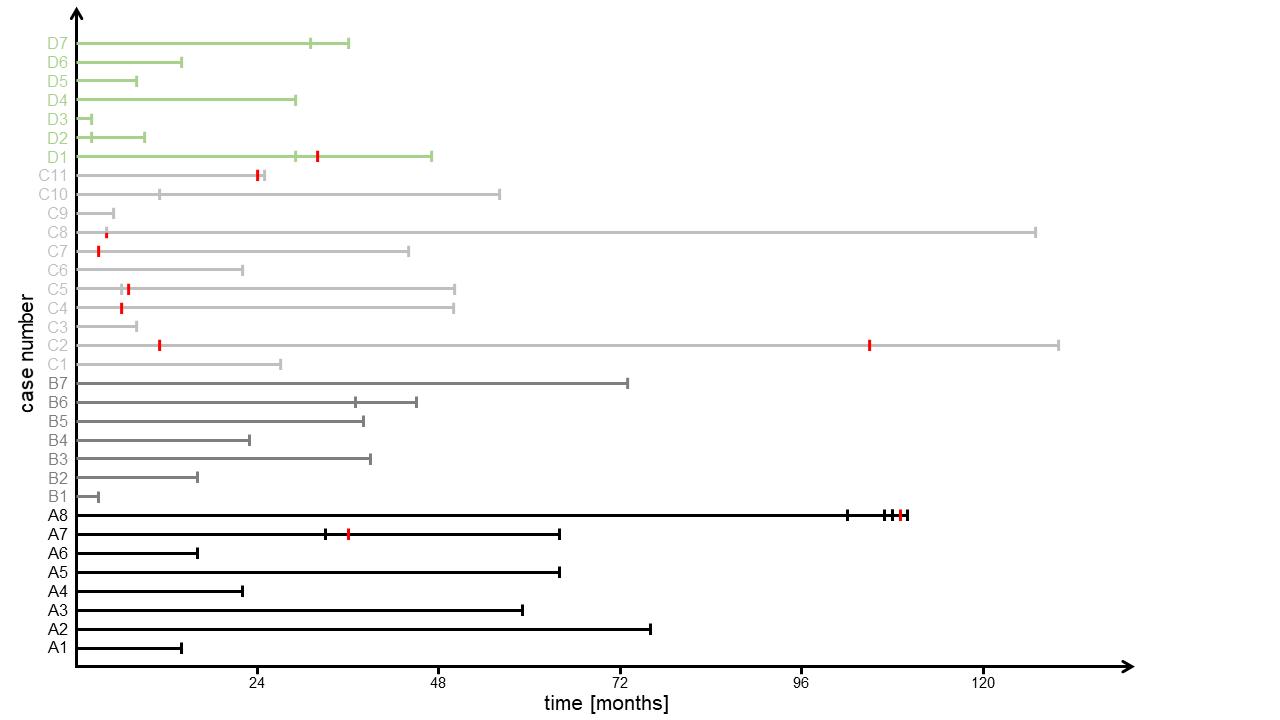
**

**Supplemental tables**

**Table S1** Data summary

| **Sample:** | **Age/Sex (M/F):** | **Cytogenetics:** | **Biopsy date (mo):** | **Diagnosis [WHO 5th edition / ICC 2022]:** | **Panel:** | **Gene (HGNC):** | **p_HGVS:** | **c_HGVS:** | **Allelic frequency:** |  |
| --- | --- | --- | --- | --- | --- | --- | --- | --- | --- | --- |
| **A: ICUS/CCUS** | |  |  |  |  |  |  |  |  |  |
| A1 | 36/M | 46, XY | + 0 | ICUS | Panel 1 | wild type | | | |  |
|  |  |  | + 14 | MDS-LB / MDS, NOS with SLD | Panel 1 | wild type | | | |  |
| A2 | 48/M | 46, XY | + 0 | ICUS | Panel 1 | wild type | | | |  |
|  |  |  | + 76 | MDS-LB post cytotoxic therapy / MDS, NOS with SLD, therapy related | Panel 1 | wild type | | | |  |
| A3 | 63/M | 46, XY | + 0 | ICUS | Panel 2 | wild type | | | |  |
|  |  |  | + 59 | MDS-LB / MDS, NOS with MLD | Panel 2 | wild type | | | |  |
| A4 | 71/F | 46, XX | + 0 | ICUS | Panel 2 | *DNMT3A* | p.R326H | c.977G>A | 11% |  |
|  |  |  |  |  |  | *SF3B1* | p.K700E | c.2098A>G | 33% |  |
|  |  |  | + 22 | MDS-*SF3B1* | Panel 2 | *DNMT3A* | p.R326H | c.977G>A | 11% |  |
|  |  |  |  |  |  | *SF3B1* | p.K700E | c.2098A>G | 40% |  |
| A5 | 75/M | 46, XY | + 0 | ICUS | Panel 1 | *KRAS* | p.A146V | c.437C>T | 1% |  |
|  |  |  |  |  |  | *TET2* | p.R1302G | c.3904A>G | 45% |  |
|  |  |  |  |  |  | *TET2* | p.L1418Q | c.4253T>A | 1% |  |
|  |  |  | + 64 | MDS-LB / MDS, NOS with MLD | Panel 2 | *NRAS* | p.G13R | c.37G>C | 4% |  |
|  |  |  |  |  |  | *KRAS* | p.A146V | c.437C>T | 9% |  |
|  |  |  |  |  |  | *TET2* | p.R1302G | c.3904A>G | 61% |  |
|  |  |  |  |  |  | *TET2* | p.L1418Q | c.4253T>A | 6% |  |
| A6 | 55/M | 46, XY, del(20)(q13.1)[≥6/46] | + 0 | ICUS | Panel 1 | *SRSF2* | p.P95H | c.284C>A | 44% |  |
|  |  |  |  |  |  | *GNAS* | p.R201H | c.602G>A | 37% |  |
|  |  |  | + 16 | AML | Panel 1 | *SRSF2* | p.P95H | c.284C>A | 23% |  |
|  |  |  |  |  |  | *GNAS* | p.R201H | c.602G>A | 26% |  |
| A7 | 55/F | +0 month: 46, XX | + 0 | ICUS | Panel 1 | *DNMT3A* | p.R882C | c.2644C>T | 35% |  |
|  |  |  |  |  |  | *IDH1* | p.R132C | c.394C>T | 16% |  |
|  |  |  | + 33 | MDS-IB2 in progress to AML / MDS/AML in progression to AML | Panel 1 | *DNMT3A* | p.R882C | c.2644C>T | 37% |  |
|  |  |  |  |  |  | *IDH1* | p.R132C | c.394C>T | 30% |  |
|  |  | +64 month: 45, X, -X or -7 [6] | + 64 | AML | Panel 1 | *DNMT3A* | p.R882C | c.2644C>T | 45% |  |
|  |  |  |  |  |  | *IDH1* | p.R132C | c.394C>T | 43% |  |
|  |  |  |  |  |  | *SRSF2* | p.P95L | c.284C>T | 4% |  |
|  |  |  |  |  |  | *RUNX1* | p.S356Hfs | c.1059_1065dup | 7% |  |
|  |  |  |  |  |  | *DNMT3A* | p.V328F | c.982G>T | 25% |  |
| A8 | 54/F | 46, XX | + 0 | ICUS | Oncomine Myeloid Research | *IDH2* | p.R140Q | c.419G>A | 46% |  |
|  |  |  |  |  |  | *SRSF2* | p.P95L | c.284C>T | 35% |  |
|  |  |  | + 102 | AML | Panel 1 | *IDH2* | p.R140Q | c.419G>A | 46% |  |
|  |  |  |  |  |  | *SRSF2* | p.P95L | c.284C>T | 27% |  |
|  |  |  |  |  |  | *NPM1* | p.W288Cfs | c.860_863dup | 36% |  |
|  |  |  |  |  |  | *FLT3* | p.I836del | c.2508_2510del | 25% |  |
|  |  |  |  |  |  | *MPL* | p.W515L | c.1544G>T | 5% |  |
|  |  |  | + 107 | Residual AML | Oncomine Myeloid Research | *IDH2* | p.R140Q | c.419G>A | 46% |  |
|  |  |  |  |  |  | *SRSF2* | p.P95L | c.284C>T | 27% |  |
|  |  |  |  |  |  | *MPL* | p.W515L | c.1544G>T | 5% |  |
|  |  |  | + 108 | Residual AML | Oncomine Myeloid Research | *IDH2* | p.R140Q | c.419G>A | 42% |  |
|  |  |  |  |  |  | *SRSF2* | p.P95L | c.284C>T | 31% |  |
|  |  |  |  |  |  | *MPL* | p.W515L | c.1544G>T | 43% |  |
|  |  |  | + 110 | Reactive | Oncomine Myeloid Research | *IDH2* | p.R140Q | c.419G>A | 1% |  |
|  |  |  |  |  |  | *SRSF2* | p.P95L | c.284C>T | 2% |  |
|  |  |  |  |  |  | *MPL* | p.W515L | c.1544G>T | 1% |  |
| **B: low grade MDS** | | |  |  |  |  |  |  |  | |
| B1 | 62/M | 46, XY, -Y [3/27] | + 0 | MDS-LB / MDS, NOS with SLD | Panel 2 | wild type | | | |  |
|  |  |  | + 3 | MDS-LB / MDS, NOS with SLD | Panel 2 | wild type | | | |  |
| B2 | 58/M | 46, XY | + 0 | MDS-LB / MDS, NOS with MLD | Panel 1 | wild type | | | |  |
|  |  |  | + 16 | MDS-LB / MDS, NOS with MLD | Panel 1 | wild type | | | |  |
| B3 | 84/M | 46, XY (13q14 del) | + 0 | MDS-LB post cytotoxic therapy / MDS, NOS with MLD, therapy related | Panel 2 | *TP53* | p.R273H | c.818G>A | 39% |  |
|  |  |  | + 39 | MDS-LB post cytotoxic therapy / MDS, NOS with MLD, therapy related | Panel 2 | *TP53* | p.R273H | c.818G>A | 52% |  |
| B4 | 75/M | N/A | + 0 | MDS-LB / MDS, NOS with MLD | Panel 2 | *ASXL1* | p.L823*fs | c.2468del | 68% |  |
|  |  |  | + 23 | MDS-LB / MDS, NOS with MLD | Panel 2 | *ASXL1* | p.L823*fs | c.2468del | 59% |  |
| B5 | 68/M | 47, XY +12 stimulated [25] | + 0 | MDS-LB / MDS, NOS with SLD | Panel 2 | *SRSF2* | p.P95_R102del | c.284_307del | 37% |  |
|  |  |  |  |  |  | *TET2* | p.N1610Ifs | c.4827del | 3% |  |
|  |  |  | + 38 | MDS-LB / MDS, NOS with MLD | Panel 2 | *SRSF2* | p.P95_R102del | c.284_307del | 34% |  |
|  |  |  |  |  |  | *TET2* | p.N1610Ifs | c.4827del | 11% |  |
| B6 | 60/M | 46, XY | + 0 | MDS-*SF3B1* | Panel 2 | *SF3B1* | p.R625C | c.1873C>T | 42% |  |
|  |  |  |  |  |  | *TET2* | p.H1904R | c.5711A>G | 75% |  |
|  |  |  |  |  |  | *TET2* | p.R1216* | c.3646C>T | 2% |  |
|  |  |  | + 37 | MDS-*SF3B1* | Panel 1 | *SF3B1* | p.R625C | c.1873C>T | 44% |  |
|  |  |  |  |  |  | *TET2* | p.H1904R | c.5711A>G | 81% |  |
|  |  |  |  |  |  | *TET2* | p.R1216* | c.3646C>T | 5% |  |
|  |  |  | + 45 | MDS-*SF3B1* | Panel 1 | *SF3B1* | p.R625C | c.1873C>T | 42% |  |
|  |  |  |  |  |  | *TET2* | p.H1904R | c.5711A>G | 85% |  |
|  |  |  |  |  |  | *TET2* | p.R1216* | c.3646C>T | 4% |  |
| B7 | 70/M | 46, XY | + 0 | MDS-*SF3B1* | Panel 1 | *CSF3R* | p.E815K | c.2443G>A | 45% |  |
|  |  |  |  |  |  | *DNMT3A* | p.S770L | c.2309C>T | 13% |  |
|  |  |  |  |  |  | *DNMT3A* | p.G646E | c.1937G>A | 2% |  |
|  |  |  |  |  |  | *SF3B1* | p.K700E | c.2098A>G | 24% |  |
|  |  |  |  |  |  | *SF3B1* | p.K666N | c.1998G>T | 10% |  |
|  |  |  |  |  |  | *ASXL1* | p.P920Tfs | c.2757dup | 3% |  |
|  |  |  | + 73 | MDS-IB2 / MDS/AML | Panel 1 | *CSF3R* | p.E815K | c.2443G>A | 47% |  |
|  |  |  |  |  |  | *DNMT3A* | p.S770L | c.2309C>T | 38% |  |
|  |  |  |  |  |  | *DNMT3A* | p.G646E | c.1937G>A | 36% |  |
|  |  |  |  |  |  | *SF3B1* | p.K700E | c.2098A>G | 2% |  |
|  |  |  |  |  |  | *SF3B1* | p.K666N | c.1998G>T | 37% |  |
| **C: high grade MDS** | | |  |  |  |  |  |  |  | |
| C1 | 68/M | N/A | + 0 | MDS-IB2 / MDS/AML | Panel 2 | *IDH1* | p.R132H | c.395G>A | 39% |  |
|  |  |  |  |  |  | *ASXL1* | p.E773Rfs | c.2316del | 34% |  |
|  |  |  | + 27 | MDS-LB / MDS, NOS with MLD | Panel 2 | *IDH1* | p.R132H | c.395G>A | 17% |  |
|  |  |  |  |  |  | *ASXL1* | p.E773Rfs | c.2316del | 11% |  |
| C2 | 65/M | 47, XY + 8 [22] | + 0 | MDS-IB1 / MDS-EB | Panel 1 | *ASXL1* | p.E635Rfs | c.1887_1910del | 3% |  |
|  |  |  | + 130 | MDS-IB1 / MDS-EB | Panel 2 | *ASXL1* | p.E635Rfs | c.1887_1910del | 19% |  |
| C3 | 77/M | 46, XY | + 0 | MDS-IB1 / MDS-EB | Panel 1 | *SRSF2* | p.P95H | c.284C>A | 53% |  |
|  |  |  |  |  |  | *ASXL1* | p.R693* | c.2077C>T | 32% |  |
|  |  |  |  |  |  | *RUNX1* | p.R204Q | c.611G>A | 15% |  |
|  |  |  |  |  |  | *RUNX1** | p.L112Q | c.335T>A | 11% |  |
|  |  |  |  |  |  | *STAG2** | p.R604* | c.1810C>T | 33% |  |
|  |  |  | + 8 | MDS-IB1 / MDS-EB | Panel 1 | *SRSF2* | p.P95H | c.284C>A | 46% |  |
|  |  |  |  |  |  | *ASXL1* | p.R693* | c.2077C>T | 20% |  |
|  |  |  |  |  |  | *RUNX1* | p.R204Q | c.611G>A | 42% |  |
|  |  |  |  |  |  | *RUNX1** | p.L112Q | c.335T>A | 11% |  |
|  |  |  |  |  |  | *STAG2** | p.R604* | c.1810C>T | 80% |  |
| C4 | 65/F | 46, XX | + 0 | MDS-IB2 / MDS/AML | Panel 2 | *RUNX1* | p.R166* | c.496C>T | 15% |  |
|  |  |  |  |  |  | *TET2* | p.Y867H | c.2599T>C | 51% |  |
|  |  |  |  |  |  | *TET2* | p.P1723S | c.5167C>T | 49% |  |
|  |  |  | + 50 | MDS-IB2 in progress to AML / MDS/AML in progression to AML | Panel 2 | *RUNX1* | p.R166* | c.496C>T | 9% |  |
|  |  |  |  |  |  | *TET2* | p.Y867H | c.2599T>C | 15% |  |
|  |  |  |  |  |  | *TET2* | p.P1723S | c.5167C>T | 13% |  |
| C5 | 59/F | 46, XX | + 0 | MDS-IB1 / MDS-EB | Panel 1 | *DNMT3A* | p.L650Q | c.1949T>A | 10% |  |
|  |  |  |  |  |  | *TET2* | p.C1271Wfs | c.3812dup | 31% |  |
|  |  |  |  |  |  | *TET2* | p.Q1834Rfs | c.5500del | 43% |  |
|  |  |  |  |  |  | *RUNX1* | p.Y140Sfs | c.419_449del | 7% |  |
|  |  |  | + 6 | MDS-IB2 in progress to AML / MDS/AML in progression to AML | Panel 1 | *DNMT3A* | p.L650Q | c.1949T>A | 4% |  |
|  |  |  |  |  |  | *TET2* | p.C1271Wfs | c.3812dup | 36% |  |
|  |  |  |  |  |  | *TET2* | p.Q1834Rfs | c.5500del | 55% |  |
|  |  |  |  |  |  | *RUNX1* | p.Y140Sfs | c.419_449del | 11% |  |
|  |  |  | + 50 | MDS-LB / MDS, NOS with MLD | Panel 1 | *TET2* | p.C1271Wfs | c.3812dup | 24% |  |
|  |  |  |  |  |  | *TET2* | p.Q1834Rfs | c.5500del | 29% |  |
|  |  |  |  |  |  | *RUNX1* | p.Y140Sfs | c.419_449del | 6% |  |
|  |  |  |  |  |  | *KRAS* | p.A146P | c.436G>C | 4% |  |
| C6 | 56/F | 46, XX; del(5q), del(17) | + 0 | MDS-IB2 / MDS/AML | Panel 1 | wild type | | | |  |
|  |  |  | + 22 | AML | Panel 1 | wild type | | | |  |
| C7 | 44/M | + 0 month: 46,XY | + 0 | MDS-IB1 / MDS-EB | Panel 1 | *IDH2* | p.R140Q | c.419G>A | 3% |  |
|  |  | + 44 month: 46,XY [20];(46,XX [9] (allogenic fraction)) | + 44 | AML | Panel 1 | *TET2* | p.Y867H | c.2599T>C | 12% |  |
|  |  |  |  |  |  | *TET2* | p.P1723S | c.5167C>T | 8% |  |
|  |  |  |  |  |  | *IDH2* | p.R140Q | c.419G>A | 32% |  |
| C8 | 54/M | 46, XY | + 0 | MDS-IB2 / MDS/AML | Panel 1 | *DNMT3A* | p.T834A | c.2500A>G | 4% |  |
|  |  |  |  |  |  | *SF3B1* | p.K700E | c.2098A>G | 16% |  |
|  |  |  |  |  |  | *RUNX1* | p.R320* | c.958C>T | 4% |  |
|  |  |  | + 4 | MDS-IB2 / MDS/AML | Panel 1 | *DNMT3A* | p.T834A | c.2500A>G | 4% |  |
|  |  |  |  |  |  | *SF3B1* | p.K700E | c.2098A>G | 22% |  |
|  |  | + 127 month: 46,XY del(5) (q13q33), del(15) (q11.2q15) [>10] |  |  |  | *RUNX1* | p.R320* | c.958C>T | 7% |  |
|  |  |  | + 127 | AML | Panel 1 | *SF3B1* | p.K700E | c.2098A>G | 34% |  |
| C9 | 55/M | 45, XY, del(6)(q?13), +7[23] | + 0 | MDS-IB1 post cytotoxic therapy / MDS-EB therapy related | Panel 2 | *KRAS* | p.T58_E63dup | c.172_189dup | 8% |  |
|  |  |  | + 5 | AML post cytotoxic therapy / AML therapy related | Panel 2 | *KRAS* | p.T58_E63dup | c.172_189dup | 3% |  |
|  |  |  |  |  |  | *KRAS* | p.Q61H | c.183A>C | 9% |  |
| C10 | 72/M | 46, XY | + 0 | MDS-IB2 / MDS/AML | Panel 2 | *IDH2* | p.R140Q | c.419G>A | 44% |  |
|  |  |  |  |  |  | *SRSF2* | p.P95L | c.284C>T | 42% |  |
|  |  |  |  |  |  | *TET2* | p.R1516* | c.4546C>T | 8% |  |
|  |  |  | + 11 | MDS-IB2 in progress to AML / MDS/AML in progression to AML | Panel 2 | *IDH2* | p.R140Q | c.419G>A | 47% |  |
|  |  |  |  |  |  | *SRSF2* | p.P95L | c.284C>T | 37% |  |
|  |  |  |  |  |  | *TET2* | p.R1516* | c.4546C>T | 7% |  |
|  |  |  |  |  |  | *KRAS* | p.A146T | c.436G>A | 6% |  |
|  |  |  | + 56 | AML | Oncomine Myeloid Research | *IDH2* | p.R140Q | c.419G>A | 47% |  |
|  |  |  |  |  |  | *SRSF2* | p.P95L | c.284C>T | 37% |  |
|  |  |  |  |  |  | *KRAS* | p.A146T | c.436G>A | 4% |  |
|  |  |  |  |  |  | *NRAS* | p.A146T | c.436G>A | 24% |  |
| C11 | 68/M | 46, XY | + 0 | MDS-IB1 / MDS-EB | Panel 2 | *SRSF2* | p.P95L | c.284C>T | 33% |  |
|  |  |  | + 25 | AML | Panel 2 | *SRSF2* | p.P95L | c.284C>T | 24% |  |
|  |  |  |  |  |  | *SETBP1* | p.G870S | c.2608G>A | 41% |  |
| **D: MDS/MPN** | | |  |  |  |  |  |  |  | |
| D1 | 62/M | 46, XY | + 0 | MDS/MPN, NOS | Panel 1 | *TET2* | p.R1261H | c.3782G>A | 43% |  |
|  |  |  |  |  |  | *SRSF2* | p.P95H | c.284C>A | 44% |  |
|  |  |  | + 29 | AML | Panel 1 | *TET2* | p.R1261H | c.3782G>A | 88% |  |
|  |  |  |  |  |  | *SRSF2* | p.P95H | c.284C>A | 49% |  |
|  |  |  |  |  |  | *RUNX1* | p.R166* | c.496C>T | 44% |  |
|  |  |  |  |  |  | *RUNX1* | p.S167Kfs | c.497_498insGA | 45% |  |
|  |  |  | + 47 | AML | Panel 1 | *TET2* | p.R1261H | c.3782G>A | 9% |  |
|  |  |  |  |  |  | *TET2* | p.P174H | c.521C>A | 37% |  |
|  |  |  |  |  |  | *SRSF2* | p.P95H | c.284C>A | 11% |  |
|  |  |  |  |  |  | *RUNX1* | p.R162K | c.485G>A | 11% |  |
|  |  |  |  |  |  | *RUNX1* | p.A149Rfs | c.444_445insCGGCATAGGGAATGCTACC | 10% |  |
| D2 | 59/M | 47,XY +8 [25] | + 0 | CMML-2 | Panel 1 | *SRSF2* | p.P95H | c.284C>A | 46% |  |
|  |  |  | + 2 | CMML-1 | Panel 1 | *SRSF2* | p.P95H | c.284C>A | 43% |  |
|  |  |  | + 9 | CMML-1 | Panel 1 | *SRSF2* | p.P95H | c.284C>A | 49% |  |
|  |  |  |  |  |  | *SETBP1* | p.D868N | c.2602G>A | 4% |  |
|  |  |  |  |  |  | *STAG2* | p.I256Mfs | c.768del | 25% |  |
| D3 | 66/M | N/A | + 0 | CMML-1 | Panel 1 | *KIT* | p.D816V | c.2447A>T | 13% |  |
|  |  |  |  |  |  | *TET2* | p.C1271Wfs | c.3812_3813insG | 45% |  |
|  |  |  |  |  |  | *TET2* | p.R1543* | c.4627A>T | 52% |  |
|  |  |  |  |  |  | *CBL* | p.R420Q | c.1259G>A | 5% |  |
|  |  |  |  |  |  | *SRSF2* | p.P95H | c.284C>A | 47% |  |
|  |  |  | + 2 | CMML-1 | Panel 1 | *KIT* | p.D816V | c.2447A>T | 13% |  |
|  |  |  |  |  |  | *TET2* | p.C1271Wfs | c.3812_3813insG | 45% |  |
|  |  |  |  |  |  | *TET2* | p.R1543* | c.4627A>T | 43% |  |
|  |  |  |  |  |  | *CBL* | p.R420Q | c.1259G>A | 11% |  |
|  |  |  |  |  |  | *SRSF2* | p.P95H | c.284C>A | 45% |  |
| D4 | 55/M | 46, XY | + 0 | CMML-1 | Panel 1 | *NRAS* | p.T58I | c.173C>T | 35% |  |
|  |  |  |  |  |  | *TET2* | p.E1186* | c.3556G>T | 44% |  |
|  |  |  |  |  |  | *TP53* | p.C275Y | c.824G>A | 42% |  |
|  |  |  |  |  |  | *SRSF2* | p.P95R | c.284C>G | 44% |  |
|  |  |  |  |  |  | *RUNX1* | p.P113A | c.337C>G | 44% |  |
|  |  |  | + 29 | CMML-1 | Panel 1 | *NRAS* | p.T58I | c.173C>T | 4% |  |
|  |  |  |  |  |  | *TET2* | p.E1186* | c.3556G>T | 45% |  |
|  |  |  |  |  |  | *TP53* | p.C275Y | c.824G>A | 36% |  |
|  |  |  |  |  |  | *SRSF2* | p.P95R | c.284C>G | 38% |  |
|  |  |  |  |  |  | *RUNX1* | p.P113A | c.337C>G | 44% |  |
|  |  |  |  |  |  | *CBL* | p.Y371H | c.1111T>C | 3% |  |
| D5 | 66/M | N/A | + 0 | CMML-2 | Panel 1 | *TET2* | p.Q769Sfs | c.2305del | 45% |  |
|  |  |  |  |  |  | *TET2* | p.E1357D | c.4071G>T | 45% |  |
|  |  |  |  |  |  | *TET2* | p.R1359C | c.4075C>T | 45% |  |
|  |  |  |  |  |  | *SRSF2* | p.P95_R102del | c.284_307del | 44% |  |
|  |  |  |  |  |  | *RUNX1* | p.S141L | c.422C>T | 44% |  |
|  |  |  | + 8 | CMML-2 | Panel 1 | *TET2* | p.Q769Sfs | c.2305del | 46% |  |
|  |  |  |  |  |  | *TET2* | p.E1357D | c.4071G>T | 46% |  |
|  |  |  |  |  |  | *TET2* | p.R1359C | c.4075C>T | 46% |  |
|  |  |  |  |  |  | *SRSF2* | p.P95_R102del | c.284_307del | 49% |  |
|  |  |  |  |  |  | *RUNX1* | p.S141L | c.422C>T | 45% |  |
| D6 | 75/M | 46, XY | + 0 | CMML-1 | Panel 1 | *NRAS* | p.G13V | c.38G>T | 30% |  |
|  |  |  |  |  |  | *TET2* | p.S1023Rfs | c.3069del | 47% |  |
|  |  |  |  |  |  | *TET2* | p.I1160F | c.3478A>T | 47% |  |
|  |  |  |  |  |  | *JAK2* | p.V617F | c.1849G>T | 30% |  |
|  |  |  |  |  |  | *SRSF2* | p.P95L | c.284C>T | 38% |  |
|  |  |  |  |  |  | *RUNX1* | p.Y380Pfs | c.1135_1136dup | 12% |  |
|  |  |  |  |  |  | *RUNX1* | p.A338P | c.1011del | 9% |  |
|  |  |  | + 14 | CMML-2 | Panel 1 | *NRAS* | p.G13V | c.38G>T | 25% |  |
|  |  |  |  |  |  | *TET2* | p.S1023Rfs | c.3069del | 47% |  |
|  |  |  |  |  |  | *TET2* | p.I1160F | c.3478A>T | 47% |  |
|  |  |  |  |  |  | *JAK2* | p.V617F | c.1849G>T | 39% |  |
|  |  |  |  |  |  | *SRSF2* | p.P95L | c.284C>T | 37% |  |
|  |  |  |  |  |  | *RUNX1* | p.Y380Pfs | c.1135_1136dup | 17% |  |
|  |  |  |  |  |  | *RUNX1* | p.A338P | c.1011del | 16% |  |
| D7 | 74/M | + 0 month: 46, XY | + 0 | CMML-1 (staging biopsy, retrospective reclassification) | Panel 1 | *TET2* | p.Q644* | c.1930C>T | 37% |  |
|  |  |  |  |  |  | *TET2* | p.G1282D | c.3845G>A | 41% |  |
|  |  |  |  |  |  | *ASXL1** | p.R693* | c.2077C>T | 4% |  |
|  |  |  |  |  |  | *ZRSR2* | p.E94Rfs | c.279del | 2% |  |
|  |  |  | + 31 | CMML-1 | Panel 1 | *TET2* | p.Q644* | c.1930C>T | 32% |  |
|  |  |  |  |  |  | *TET2* | p.G1282D | c.3845G>A | 32% |  |
|  |  |  |  |  |  | *ASXL1** | p.R693* | c.2077C>T | 1% |  |
|  |  |  |  |  |  | *IDH2* | p.R140Q | c.419G>A | 13% |  |
|  |  |  |  |  |  | *SRSF2* | p.P95H | c.284C>A | 15% |  |
|  |  |  |  |  |  | *ZRSR2* | p.E94Rfs | c.279del | 7% |  |
|  |  | + 39 month: 48, XY, +8,t(8;21)(q22;22), +9[16],/46,XY[38] | + 39 | AML | Panel 1 | *TET2* | p.Q644* | c.1930C>T | 41% |  |
|  |  |  |  |  |  | *TET2* | p.G1282D | c.3845G>A | 39% |  |
|  |  |  |  |  |  | *ASXL1** | p.R693* | c.2077C>T | 0.9% |  |
|  |  |  |  |  |  | *IDH2* | p.R140Q | c.419G>A | 4% |  |
|  |  |  |  |  |  | *SRSF2* | p.P95H | c.284C>A | 5% |  |
|  |  |  |  |  |  | *ZRSR2* | p.E94Rfs | c.279del | 3% |  |
|  | | |  |  |  |  |  |  |  | |

* in the column "gene": Variants that have been validated

**Table S2** Detailed coverage of panel 1

| **Gene** | **Transcript** | **Position (GRCh37/hg19)** | **Exon(s)** | **Amplicons** | **Coverage of CDS (%)** |
| --- | --- | --- | --- | --- | --- |
| ***ASXL1*** | NM_015338 | chr20: 31021090 - 31021725 | 11 | 7 | - |
|  |  | chr20: 31022232 - 31025150 | 12 | 32 | - |
| ***BRAF*** | NM_004333 | chr7: 140453100 - 140453195 | 13 | 1 | - |
|  |  | chr7: 140481390 - 140481500 | 15 | 2 | - |
| ***CALR*** | NM_004343 | chr19: 13054526 - 13054700 | 9 | 3 | - |
| ***CBL*** | NM_005188 | chr11: 119148465 - 119148555 | 7 | 1 | - |
|  |  | chr11: 119148875 - 119149010 | 8 | 2 | - |
|  |  | chr11: 119149215 - 119149425 | 9 | 3 | - |
| ***CEBPA*** | NM_004364 | chr19: 33792146 - 33793455 | CDS | 10 | 78% |
| ***CSF3R*** | NM_000760 | chr1: 36932080 - 36932330 | 17 | 4 | - |
|  |  | chr1: 36933430 - 36933445 | 14 | 1 | - |
| ***DNMT3A*** | NM_022552 | chr2: 25457125 - 25536909 | CDS | 52 | 100% |
| ***ETNK1*** | NM_001039481 | chr12: 22797185 - 22797200 | 3 | 1 | - |
| ***EZH2*** | NM_004456 | chr7: 148504653 - 148544408 | CDS | 37 | 100% |
| ***FLT3*** | NM_004119 | chr13: 28592635 - 28592650 | 14 | 1 | - |
|  |  | chr13: 28608219 - 28608351 | 20 | 2 | - |
| ***GNAS*** | NM_000516 | chr20: 57484400 - 57484480 | 8 | 1 | - |
|  |  | chr20: 57484570 - 57484640 | 9 | 1 | - |
| ***HRAS*** | NM_005343 | chr11: 533805 - 533935 | 3 | 2 | - |
|  |  | chr11: 534215 - 534310 | 2 | 2 | - |
| ***IDH1*** | NM_005896 | chr2: 209113108 - 209113120 | 4 | 1 | - |
| ***IDH2*** | NM_002168 | chr15: 90631830 - 90631940 | 4 | 2 | - |
| ***IKZF1*** | NM_006060 | chr7: 50358636 - 50468407 | CDS | 28 | 100% |
| ***JAK2*** | NM_004972 | chr9: 5069924 - 5070053 | 12 | 2 | - |
|  |  | chr9: 5073679 - 5073801 | 14 | 1 | - |
| ***KIT*** | NM_000222 | chr4: 55589750 - 55589780 | 8 | 1 | - |
|  |  | chr4: 55599320 - 55599322 | 17 | 1 | - |
| ***KRAS*** | NM_033360 | chr12: 25378550 - 25378660 | 4 | 2 | - |
|  |  | chr12: 25380270 - 25380290 | 3 | 1 | - |
|  |  | chr12: 25398275 - 25398290 | 2 | 1 | - |
| ***MPL*** | NM_005373 | chr1: 43814930 - 43815035 | 10 | 2 | - |
| ***NPM1*** | NM_002520 | chr5: 170814943 - 170837600 | CDS | 19 | 100% |
| ***NRAS*** | NM_002524 | chr1: 115252195 - 115252295 | 4 | 1 | - |
|  |  | chr1: 115256520 - 115256540 | 3 | 1 | - |
|  |  | chr1: 115258740 - 115258750 | 2 | 1 | - |
| ***RUNX1*** | NM_001754 | chr21: 36164294 - 36421237 | CDS | 24 | 99% |
| ***SETBP1*** | NM_015559 | chr18: 42531900 - 42531920 | 4 | 1 | - |
| ***SF3B1*** | NM_012433 | chr2: 198266465 - 198266615 | 16 | 4 | - |
|  |  | chr2: 198266705 - 198266855 | 15 | 3 | - |
|  |  | chr2: 198267275 - 198267555 | 14 | 3 | - |
| ***SRSF2*** | NM_003016 | chr17: 74732881 - 74733000 | 1 | 1 | 94% |
| ***STAG2*** | NM_006603 | chrX: 123156465 - 123234506 | CDS | 71 | 100% |
| ***STAT3*** | NM_139276 | chr17: 40474300 - 40474512 | 21 | 2 | - |
|  |  | chr17: 40475022 - 40475161 | 20 | 2 | - |
| ***TET2*** | NM_017628 | chr4: 106155065 - 106197684 | CDS | 68 | 100% |
| ***TP53*** | NM_000546 | chr17: 7572847 - 7579960 | CDS | 23 | 100% |
| ***U2AF1*** | NM_006758 | chr21: 44514770 - 44514790 | 6 | 1 | - |
|  |  | chr21: 44524440 - 44524470 | 2 | 1 | - |
| ***ZRSR2*** | NM_005089 | chrX: 15808513 - 15841397 | CDS | 24 | - |

**Table S3** Detailed coverage of panel 2

| **Gene** | **Transcript** | **Position (GRCh37/hg19)** | **Exon(s)** | **Amplicons** | **Coverage of CDS (%)** |
| --- | --- | --- | --- | --- | --- |
| ***ASXL1*** | NM_015338 | chr20: 31,021,082 – 31,021,725 | 11 | 7 | - |
|  |  | chr20: 31,022,230 – 31,024,815 | 12 | 27 | - |
|  |  | chr20: 31,024,841 – 31,025,141 | 12 | 4 | - |
| ***CBL*** | NM_005188 | chr11: 119,148,462 – 119,149,428 | 7+8+9 | 6 | - |
| ***DNMT3A*** | NM_022552 | chr2: 25,457,148 – 25,536,853 | CDS | 47 | 96% |
| ***FLT3*** | NM_004119 | chr13: 28,608,214 – 28,608,351 | 14 | 2 | - |
|  |  | chr13: 28,592,608 – 28,592,712 | 20 | 1 | - |
| ***IDH1*** | NM_005896 | chr2: 209,113,088 – 209,113, 194 | 4 | 1 | - |
| ***IDH2*** | NM_002168 | chr15: 90,631,814– 90,631,955 | 4 | 2 | - |
| ***KRAS*** | NM_033360 | chr12: 25,398,203 – 25,398, 310 | 2 | 1 | - |
|  |  | chr12: 25,380,260 – 25,380, 337 | 3 | 1 | - |
|  |  | chr12: 25,378,543 – 25,378,663 | 4 | 2 | - |
| ***NRAS*** | NM_002524 | chr1: 115,258,687 – 115,258,781 | 2 | 1 | - |
|  |  | chr1: 115,256,463 – 115,256,578 | 3 | 1 | - |
|  |  | chr1: 115,252,185 – 115,252,309 | 4 | 1 | - |
| ***RHOA*** | NM_001664 | chr3: 49,412,916 – 49,413,022 | 2 | 1 | - |
| ***RUNX1*** | NM_001754 | chr21: 36,164,432 – 36,421,196 | CDS | 20 | 89% |
| ***SETBP1*** | NM_015559 | chr18: 42,531,789 – 42,531, 951 | 4 | 2 | - |
| ***SF3B1*** | NM_012433 | chr2: 198,266,704 – 198,267,554 | 14+15 | 6 | - |
| ***SRSF2*** | NM_003016 | chr17: 74,732,889 – 74,733,028 | 1 | 1 | - |
| ***TET2*** | NM_001127208 | chr4: 106,155,100 – 106,197,676 | CDS | 68 | 99% |
| ***TP53*** | NM_000546 | chr17: 7,572,927– 7,579,912 | CDS | 22 | 98% |
| ***U2AF1*** | NM_006758 | chr21: 44,524,432 – 44,524,508 | 2 | 1 | - |
|  |  | chr21: 44,514,760 – 44,514,832 | 6 | 1 | - |
| ***ZRSR2*** | NM_005089 | chrX: 15,808,619 – 15,841,365 | CDS | 22 | 93% |

**Table S4** Primer for variant validation

| **Gene** | **Chromosomal location:** | **c_HGVS:** | **p_HGVS:** | **Primer:** | **Sequence (5' to 3'):** |
| --- | --- | --- | --- | --- | --- |
| *SETBP1* | chr18:42531950 | c.2645C>T | p.A882V | Forward | CCATCTCATCCCTGCGTGTCTCCGACTCAGTTGGCATCTCGATCCCACAGTGAGGAGACGATC |
|  |  |  |  | trP1 Forward | CCTCTCTATGGGCAGTCGGTGATCCCACAGTGAGGAGACGATC |
|  |  |  |  | Reverse | CCATCTCATCCCTGCGTGTCTCCGACTCAGTTGGCATCTCGATTGTCCAGGGAGCAGAAATCA |
|  |  |  |  | trP1 Reverse | CCTCTCTATGGGCAGTCGGTGATTGTCCAGGGAGCAGAAATCA |
| *ASXL1* | chr20:31022592 | c.2077C>T | p.R693* | Forward | CCATCTCATCCCTGCGTGTCTCCGACTCAGCTAGGACATTCGATCGAGCACCCCTGGAAAGT |
|  |  |  |  | trP1 Forward | CCTCTCTATGGGCAGTCGGTGATCGAGCACCCCTGGAAAGT |
|  |  |  |  | Reverse | CCATCTCATCCCTGCGTGTCTCCGACTCAGCTAGGACATTCGATCTAGCTCTGGACATGGCAGT |
|  |  |  |  | trP1 Reverse | CCTCTCTATGGGCAGTCGGTGATCTAGCTCTGGACATGGCAGT |
| *RUNX1* | chr21:36259156 | c.335T>A | p.L112Q | Forward | CCATCTCATCCCTGCGTGTCTCCGACTCAGCTTCCATAACGATCCAACTTCCTCTGCTCCGT |
|  |  |  |  | trP1 Forward | CCTCTCTATGGGCAGTCGGTGATCCAACTTCCTCTGCTCCGT |
|  |  |  |  | Reverse | CCATCTCATCCCTGCGTGTCTCCGACTCAGCTTCCATAACGATTCCTCCCACCACCCTCTC |
|  |  |  |  | trP1 Reverse | CCTCTCTATGGGCAGTCGGTGATTCCTCCCACCACCCTCTC |
| *STAG2* | chrX:123197044 | c.1810C>T | p.R604* | Forward | CCATCTCATCCCTGCGTGTCTCCGACTCAGCCAGCCTCAACGATTTGCCTCAGTACTTTGATTTGGA |
|  |  |  |  | trP1 Forward | CCTCTCTATGGGCAGTCGGTGATTTGCCTCAGTACTTTGATTTGGA |
|  |  |  |  | Reverse | CCATCTCATCCCTGCGTGTCTCCGACTCAGCCAGCCTCAACGATGGAAGCATCATTACCGCCAT |
|  |  |  |  | trP1 Reverse | CCTCTCTATGGGCAGTCGGTGATGGAAGCATCATTACCGCCAT |

**Reference**

1. van Dongen JJ, Langerak AW, Bruggemann M, Evans PA, Hummel M, Lavender FL, Delabesse E, Davi F, Schuuring E, Garcia-Sanz R, van Krieken JH, Droese J, Gonzalez D, Bastard C, White HE, Spaargaren M, Gonzalez M, Parreira A, Smith JL, Morgan GJ, Kneba M, Macintyre EA (2003) Design and standardization of PCR primers and protocols for detection of clonal immunoglobulin and T-cell receptor gene recombinations in suspect lymphoproliferations: report of the BIOMED-2 Concerted Action BMH4-CT98-3936. Leukemia 17:2257-2317. <https://doi.org/10.1038/sj.leu.2403202>
